# Cluster Analysis: A New Approach for Identification of Underlying Risk Factors and Demographic Features of First Trimester Pregnancy Women

**DOI:** 10.3390/jcm9072247

**Published:** 2020-07-15

**Authors:** Anna Karen Gárate-Escamilla, Edelmiro Garza-Padilla, Agustín Carvajal Rivera, Celina Salas-Castro, Emmanuel Andrès, Amir Hajjam El Hassani

**Affiliations:** 1 NIT Lab, Univ. Bourgogne Franche-Comte, UTBM, F-90010 Belfort, France; amir.hajjam@utbm.fr; 2 Monterrey Institute of Technology and Higher Education, Monterrey 64700, Mexico; edelmiro13@gmail.com (E.G.-P.); agustin.carvajal@itesm.mx (A.C.R.); dracelinasalas@gmail.com (C.S.-C.); 3 Service de Médecine Interne, Diabète et Maladies Métaboliques de la Clinique Médicale B, CHRU de Strasbourg, 67091 Strasbourg, France; emmanuel.andres@chru-strasbourg.fr

**Keywords:** thyroid pathology, k-means, first trimester pregnancy, pregnancy complications, pregnancy risk factors

## Abstract

Thyroid pathology is reported internationally in 5–10% of all pregnancies. The overall aim of this research was to determine the prevalence of hypothyroidism and risk factors during the first trimester screening in a Mexican patients sample. We included the records of 306 patients who attended a prenatal control consultation between January 2016 and December 2017 at the Women’s Institute in Monterrey, Mexico. The studied sample had homogeneous demographic characteristics in terms of age, weight, height, BMI (body mass index) and number of pregnancies. The presence of at least one of the risk factors for thyroid disease was observed in 39.2% of the sample. Two and three clusters were identified, in which patients varied considerably among risk factors, symptoms and pregnancy complications. Compared to Cluster 0, one or more symptoms or signs of hypothyroidism occurred, while Cluster 1 was characterized by healthier patients. When three clusters were used, Cluster 2 had a higher TSH (thyroid stimulating hormone) value and pregnancy complications. There were no significant differences in perinatal variables. In addition, high TSH levels in first trimester pregnancy are characterized by pregnancy complications and decreased newborn weight. Our findings underline the high degree of disease heterogeneity with existing pregnant hypothyroid patients and the need to improve the phenotyping of the syndrome in the Mexican population.

## 1. Introduction

Pregnancy has a profound impact on the thyroid gland and its function, making it the second most common endocrine disorder during pregnancy, after diabetes mellitus. Thyroid pathology has a variable incidence and depends on the series consulted; internationally, thyroid disorders are reported in between 5 and 10% of all pregnancies [1]. The prevalence of subclinical hypothyroidism during pregnancy is 3.0% to 5.0% [2]. The normal upper limit range of TSH during the first trimester is 2.5 mIU/L and 3.0 mIU/L for the second and third trimester [3]. Numerous risk factors for pregnancy have been reported to be associated with thyroid disease disorders, including overweigh [4], excessive salt intake [5,6,7] and high cholesterol levels [8]. The thyroid gland increases by 10% in size in countries without iodine deficiency such as Mexico. Thyroxine (T4) and triiodothyronine (T3) production increase by 50%. These physiological changes can lead to hypothyroidism in the advanced stages of pregnancy in patients with iodine deficiency who were euthyroid in early pregnancy [9]. For women with undiagnosed thyroid disease, early screening may be the ideal opportunity to allow adequate treatment. The multitude of adverse effects associated with untreated thyroid disease leads to consideration of the potential benefits of screening during preconception and pregnancy [9,10].

Hypothyroidism is an endocrinopathy characterized by an inappropriate action of thyroid hormones in the body, whose main cause is a lack of production by the thyroid gland. In countries without iodine deficiency, such as Mexico, the most common cause is autoimmune. However, we are still far from a global understanding of the problem, such as the relationship of maternal thyroid hormones with the fetus [11,12,13], the way the product develops its metabolic system from the iodine in the maternal diet [7,14], the influence this has on neuronal development [11,15] and, finally, the behavior of antithyroid antibodies during pregnancy [16], especially in the puerperium [17].

Subclinical hypothyroidism has not been clearly associated with adverse maternal–fetal outcomes such as overt hypothyroidism. The consequences of the former have been poorly defined, although most studies report an association between them and adverse pregnancy outcomes [3,9]. Abalovich and coworkers [18] reported that inadequate management with levothyroxine in women with manifest or subclinical hypothyroidism is associated with significant risks of miscarriage or preterm birth. However, studies have not been consistent in demonstrating these relationships. A retrospective study by Casey [19] showed that the risk of placental abruption and preterm delivery increased 2–3 times before 34 weeks of gestation in women without treatment for subclinical hypothyroidism, compared to euthyroid controls. Cleary-Goldman and coworkers [20] found no association between subclinical hypothyroidism and adverse perinatal outcomes in a cohort analysis of 10,990 women. Similarly, Männistö [21,22] also found no association in a large and retrospective cohort study.

Multiple prospective and retrospective studies have demonstrated an increased risk of complications during pregnancy [18,23] associated with a slight increase in maternal TSH levels, especially in women with positive anti-TPO antibodies. Some risks are miscarriage, preeclampsia, hypertension, baby’s brain development, hemorrhages, premature delivery, postpartum depression and low birth weight [24,25,26,27,28,29,30,31]. Only a limited number of studies have investigated the impact of levothyroxine treatment in patients with anti-TPO antibodies [3]. The randomized controlled study of Negro [23] showed a potential benefit of levothyroxine intervention at nine weeks gestation. Importantly, this study documented a reduction in adverse pregnancy outcomes only in patients with positive anti-TPO antibodies and mild hypothyroidism (defined as TSH > 2.5 mU/L). This did not provide treatment to patients with negative antibodies. It was concluded that universal screening of high TSH concentrations does not improve the results compared to a strategy focused on high-risk patients. However, despite the limitations of the available studies on levothyroxine treatment in patients with subclinical hypothyroidism, the data appear to suggest a benefit in reducing the rate of miscarriages in patients with positive anti-TPO antibodies. It is reasonable to consider levothyroxine treatment for specific subgroups of patients with subclinical hypothyroidism.

Considering the research on pregnant women in the first trimester with thyroid diseases, studies in the literature are limited. Li [32] evaluated the relationship between miscarriage and first trimester thyroid function, finding that a TSH higher than 2.5 mIU/L increased the risk for miscarriage. Hernandez and coworkers [33] found an increased risk of perinatal loss, miscarriage and premature birth for patients between 2.5 mIU/L and 4.0 mIU/L. In some countries, the authors concluded that 2.5 mIU/L is a low upper limit for first trimester pregnancy, with suggestions of an increase in the Indian population [34], the Chinese population [35], the Brazilian population (2.7 mIU/L) [36] and the Spanish population (4.72 mIU/L) [37]. Given the lack of information about the first trimester of pregnancy, this study addresses the creation of groups considering thyroid risk factors through clustering.

Cluster analysis is an unsupervised machine learning technique that aims to define subgroups of homogeneous individuals with attributes more similar to those of other groups or clusters [38,39]. Clustering is necessary to handle the interaction of multiple variables and has been used in the medical field for image processing, document classification and group creation [40]. Studies in other medical areas suggest that cluster analysis leads to a better understanding of the disease, as happens in the risk factors of coronary disease [41,42,43], chronic obstructive pulmonary disease (COPD) [44,45,46,47], asthma [48,49,50,51], tinnitus [52,53], insulin resistance in obese patients [54,55], diabetes [56,57,58], low back pain [59,60,61,62,63] and osteoarthritis [64,65,66].

Some groups found in the pregnancy-related literature are associated with hypertension [67,68], preeclampsia [69,70], fetal growth restriction [71], miscarriage [72], incidence of pregnancy termination related to demographics [73], dietary patterns [74] and birth control related to maternal education, hygiene and nutrients [75]. There are some studies related to cluster analysis and thyroid disease, such as the prevalence of thyroid diseases in children and adolescents [76], spatial distribution and risk factors related to thyroid cancer [77,78,79], and finding the optimal number of thyroid disease clusters [80,81].

However, there are few reports on clustering to better classify hypothyroidism during the first trimester of pregnancy, especially with risk factors and pregnancy complications. In this study, we applied cluster analysis to explore possible subgroups within a well-characterized population of first trimester pregnant women with hypothyroidism. This study would also help to identify potential risk factors for hypothyroidism in pregnant patients. The general objective of this research is to determine the clusters related to first trimester pregnant women with hypothyroidism in a sample of Mexican patients, with and without risk factors.

## 2. Experimental Section

### 2.1. Dataset

In this section, we describe the dataset used in the empirical testing of the first trimester pregnant women with thyroid disease (Table 1). This is a replicative, observational, cross-sectional, descriptive, retrospective study to determine the prevalence of hypothyroidism during screening of Mexican patients during the first trimester of pregnancy between January 2016 and December 2017, with and without risk factors, in a private Women’s Institute in Monterrey, Mexico. This study was the first to be conducted on a Mexican population of northern Mexico that seeks to determine the prevalence of thyroid disease during the first trimester of pregnancy screening. The inclusion criteria were the records reporting TSH values during the first trimester of pregnancy. We excluded records of patients who initiated prenatal control in the second and third trimesters, incomplete records (not having all the variables to be analysed) and foreign patient, resulting in 306 patients with 55 features. A prevalence of thyroid pathology was identified in 18% (*n* = 55) of patients, 11.8% (*n* = 36) with subclinical hypothyroidism (Table S1). If the patients had subclinical hypothyroidism, they were treated; if it was overt hypothyroidism, they were sent to the endocrinologist (although they continued to see the gynecologist).

The thyroid profiles requested during this consultation were reviewed, specifically the TSH and T4L values. From this, the patient was classified as euthyroid, with subclinical or overt hypothyroidism, or hyperthyroid according to the guidelines of the American Thyroid Association [9]. TSH values during the first trimester are between 0.1 and 2.5 mU/L in normal patients. Values with TSH greater than 2.5 mU/L without T4L alteration are diagnosed with subclinical hypothyroidism while those with decreased T4L have overt hypothyroidism. In opposite cases, decreased TSH values would indicate thyrotoxicosis. We used an upper limit of 2.5 mU/L for the following reasons: (i) The study was conducted between January 2016 and December 2017, and the Guidelines of the American Thyroid Association 2017 was published after our study began; and (ii) in Mexico, reference tables per trimester of thyroid hormone values have not yet been generated. Additional variables to analyze in each patient record will be the presence of hypertensive disease in previous pregnancies, type II diabetes and chronic hypertension. This study combined the classification of subclinical and overt hypothyroidism; additionally, no patients presented thyrotoxicosis.

As secondary information, the perinatal results of each patient were collected in retrospect: Gestational age at delivery, birth route, weight, height and gestational age by the Capurro Method of the product. This study also included the presence or absence of maternal complications, such as the development of hypertensive diseases associated with pregnancy, gestational diabetes, membranes rupture and preterm delivery. We attempted to correlate these data to determine if there are differences between patients with hypothyroidism and the rest of the population studied.

After identifying a dossier that met the inclusion criteria, it was assigned an identification number within the database and it was necessary to fill in the different variables that conform to the pathological history, risk factors for the development of thyroid diseases, thyroid pathology, pregnancy complications and perinatal results. Of the patients, 70% were over 30 years old; all patients had complete clinical data and had tested TSH.

One of the main limitations during the implementation of the protocol was the inclusion rate of 71.6%, in most cases as a result of incomplete records of relevant information, such as TSH values and perinatal results. The features that were mostly incomplete, and later removed from the study, are T4 Total, T4L, T3 Total, and Capurro. Chronical arterial hypertension and hair loss, variables associated with thyroid disease, had only one class and were also excluded from the study. A total of 49 features remained.

### 2.2. Application on the Dataset

Given a cohort of patients diagnosed with a certain disease, unsupervised machine learning models allow us to identify comorbidity clusters for that disease, which helps to define new possible risk fields. The proposed *k*-means model represents diseases in the feature space [82]. We theorize that the disease with similar characteristics would be clustered in the feature space.

To verify the effectiveness of *k*-means in identifying disease clusters, we qualitatively visualize the disease representation in the dimensional projection using centroid-based clustering. In doing so, we evaluate the potential of unsupervised machine learning in the discovery of clusters. We tested different combinations and chose the ones that generated the best visualization results for the feature space. To determine which *k* instances in the training dataset are most similar to a new entry, a Euclidean distance measure is used [83]. In addition, data must be standardized to make features comparable with the same scale. Standardization consists of transforming the features so that they have mean zero and standard deviation one [84]. We use average and standard deviation for the standardization.

### 2.3. Discovering Patient Subgroup

One question that arises in the field of gynecology is whether patients can be stratified into subgroups in which they share similar medical characteristics and risks. To discover patient subgroups, we could leverage clustering analysis on the patient feature vectors by using the rows of patient-disease. In our experiments, we tested *k*-means clustering with seven different amounts of subgroups, ranging from 2 to 8 subgroups. To evaluate these subgroups, we carried out the Within Cluster Sum of Squares (WCSS) and silhouette with squared Euclidean distance to compare the patient subgroup results. In addition to SSE and Euclidian distance, we conducted statistical analysis on the demographics and number of patient subgroups diagnoses. Our goal was to evaluate whether the patient subgroups discovered by *k*-means model could differentiate patients into a defined cohort.

#### 2.3.1. Cluster Analysis

We used Apache Spark 4.3 for cluster analysis. A *k*-means analysis was performed to find different clusters using the patient data. Feature selection, mapping features from high-dimension to low-dimension, was used to reduce the primary data. The cluster analysis carried out did not have any missing values. The main steps in the *k*-means algorithm are: (1) Randomly select initial cluster centers with the *k*-number to assign the centroids; (2) all the closest data points to the centroids will create a cluster; (3) and compute new centers for the clusters. We calculate the distance between the points and the center using the Euclidean distance. Steps 2 and 3 are repeated until the centroids stop moving. The decision of the number of *k* is made through the elbow method [85] and silhouette and verified by random forests (RF) classifier [86].

#### 2.3.2. Statistical Analysis

All statistical analyses were performed using the SPSS software package version 23.0 (SPSS Inc., Chicago, IL, USA). An analysis of each specific risk factor was performed as part of the screening for thyroid disease to determine if it was statistically significant in the sample. On the other hand, multiple comparisons were made between patients with subclinical hypothyroidism and patients without this pathology, assessing each variable within the study to determine if there is a statistically significant correlation of any of them with the pathology. Results were analyzed using descriptive statistics, means and ranges, Chi square for the crossing of nominal variables and t de Student for differences in group means. A calculated difference of *p* > 0.05 was statistically significant. Chi-squared was performed to check the significance of the binary and continuous variables, separately, between different clusters.

### 2.4. Experiments

To find the right cohort for women in the first trimester of pregnancy, we conducted four experiments using different sets of features with *k*-means: (1) We used the remaining 49 features and selected *k* = 2; (2) we performed the analysis of all features without perinatal results (weeks of Unemployment Gestation, birth route, product weight, product size and Capurro) and pregnancy complications (hypertensive disease with pregnancy, gestational diabetes, premature rupture of membrane and preterm delivery) with *k* = 2 and *k* = 3; (3) we exclusively used the thyroid pathology to create new clusters using *k* = 2; and (4) finally we used the risk factors to determine the new clusters with a *k* = 2.

For each test a classification analysis is made with RF considering the next set of features: (1) All features; (2) risk factors; (3) thyroid features; (4) symptoms or signs suggesting thyroid hypofunction; (5) all features without thyroid data; (6) all features without perinatal, pregnancy and thyroid data; and (7) all features without perinatal data and pregnancy complications.

## 3. Results

### 3.1. Determining Number of Clusters

**Figure 1:**
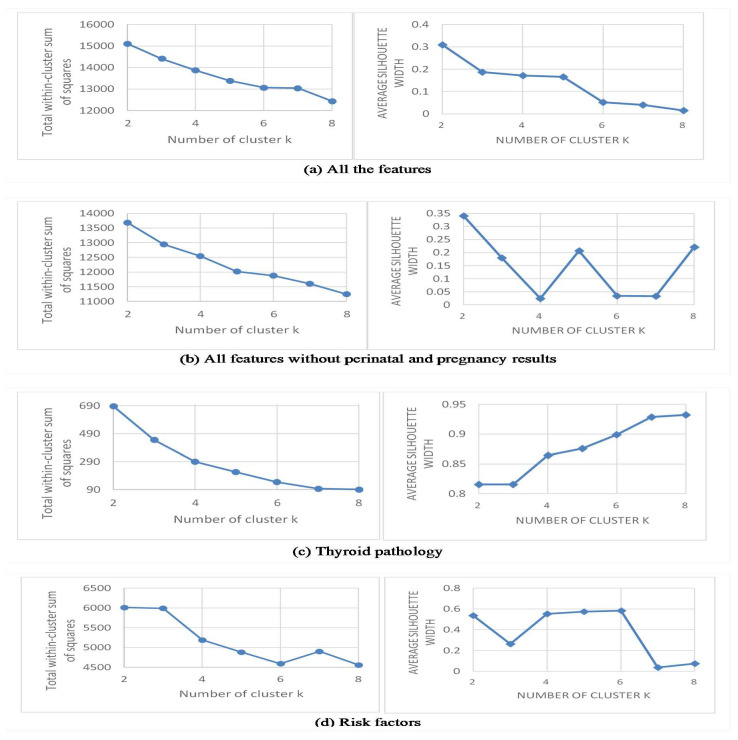
Elbow method and the average silhouette.

*k*-means requires that the number of clusters is determined in advance and supplied to the algorithm as a parameter. To measure the quality of the clusters, Figure 1 shows the elbow method and the average silhouette approach. The goal is to choose a small *k*-value that still has a low within-cluster sum of squares and a high silhouette. The results of the elbow method, the total within-cluster sum of squares, are ambiguous for (a) all features, and (b) all features without perinatal and pregnancy results. For (c) thyroid pathology the best cluster is *k* = 3 and for (d) risk factors it is *k* = 4. The highest value for the average silhouette is (a) *k* = 2 at 0.31, (b) *k* = 2 at 0.34, (c) *k* = 8 at 0.93 and (d) *k* = 6 at 0.58.

Table 2 shows the clusters, from 2 to 8, with the number of women in each one. In the case of (a) all features, after four clusters, the number of patients is small in some of the subgroups (i.e., Cluster 5 with 15 women in group 2). Based on the results provided by the silhouette, the best cluster number is *k* = 2. Similar to (a) all features, after four clusters, the (b) features without perinatal and pregnancy results start to present clusters with small values (i.e., Cluster 5 with seven women in group 4) and the best cluster number should be *k* = 2. Taking out clusters two and three, there is, for (c) thyroid pathology, a small number of women in the remaining clusters (i.e., Cluster 4 with 28 women in group 0 and 26 in group 2). The silhouette method has great results due to the small size of the subsets of the different clusters and is not reliable; based on this, we selected *k* = 2 and *k* = 3. After two clusters, for (d) risk factors, there is a small number of women (i.e., Cluster 3 with five women in group 1). According to these observations, we defined *k* = 2 as the optimal number of clusters in the data.

### 3.2. Cluster Analysis

Table S2 presented the mean and standard deviation of the features. The cluster analysis identified groups that were significantly different from each other. The anthropometric, gynecological, pathological history, risk factors, thyroid pathology and perinatal results were stratified according to phenogroup (Table 1). Key cohorts of each first-trimester pregnant women are as follows.

#### 3.2.1. All Features with *k* = 2

The cluster analysis identified two women clusters. Table 3 shows the complete baseline data for the 49 prespecified features according to the cluster.

Cluster 0 (*n* = 69) was the smallest cluster and mainly involved patients with risk factors. Women were likely to have at least one symptom or sign suggestive of thyroid hypofunction (average of 1.74); fatigue (0.48 ± 0.50) and constipation (0.41 ± 0.49) were the most prevalent features. In addition, all subjects had some risk factor involved and the presence of positive antibodies (0.06). These women had the lowest TSH level (0.06) and the highest number of diagnoses based on the thyroid profile (0.32). Furthermore, they did not present autoimmune disease, type I diabetes, or previous irradiation to the neck or head.

Cluster 1 (*n* = 237) was the largest, with >2 times more women than the other cluster. Significantly, all patients in this cluster did not have symptoms or signs suggesting thyroid hypofunction. The cluster had the highest rate of women with TSH reported (0.6) and the healthiest thyroid profile (0.14). Additionally, this cluster had the lowest levels of women with infertility (0.02) and IVF/ICSI treatments (0.01). No positive antibodies were present.

#### 3.2.2. All Features without Perinatal and Pregnancy Complication Results with *k* = 2

The cluster analysis identified two women clusters. Table 4 shows the complete baseline data for the 40 prespecified features according to the cluster.

Cluster 0 (*n* = 65) was the smallest cluster and consisted mainly of women with risk factors. It is likely that all subjects in the cluster had almost one symptom or sign suggesting thyroid hypofunction (0.85). Fatigue (0.22 ± 0.41) was the predominant feature in the presence of signs or symptoms and more than half of the women had some risk factor. They had the highest number of diagnoses based on the thyroid profile (0.38). Furthermore, Cluster 0 women were overweight (BMI of 25.96), had a higher number of miscarriages (0.29) and did not present ectopic pregnancies.

Cluster 1 (*n* = 241) was the largest, with more than two-thirds of all the women in our study. It is important to note that most subjects did not present with symptoms or signs suggestive of thyroid hypofunction and only one-third of the women had any risk factor present. Women had fewer diagnoses based on the thyroid profile (0.12). Moreover, this cluster had the lowest levels of patients with infertility (0.02) and IVF/ICSI (0.02).

#### 3.2.3. All Features without Perinatal and Pregnancy Complications Results with *k* = 3

The cluster analysis identified three women clusters. Table 5 shows the complete baseline data for the 40 prespecified features according to the cluster.

Cluster 0 (*n* = 68) was the smallest and all the women had one or more risk factors. The women had almost two symptoms or signs suggestive of thyroid hypofunction, with an average of 1.75 and a standard deviation of 1.32. Fatigue (0.47) and constipation (0.41) were the predominant features in the presence of signs or symptoms, with dry skin being the least common factor (0.04). Among the clusters, this one had the largest number of women diagnosed with thyroid profile (0.32) and on treatment (0.32). This cluster was the only one with cases of positive antibodies (0.06).

Cluster 1 (*n* = 161) was the largest, with less than half of all subjects in our study and had normal weight (BMI = 23.84). Importantly, all patients in this cluster presented no symptoms or signs suggesting thyroid hypofunction and 95% had no risk factors. Moreover, this cluster had the lowest number of women diagnosed with thyroid profile (0.09) and on TX treatment (0.08).

Cluster 2 (*n* = 77) were mainly overweight (BMI of 26.04) women between 30 and 35 years old. Gynecological values differed from those of Clusters 1 and 2. Women had more previous pregnancies (3.19 ± 1.66), vaginal deliveries (0.30 ± 0.46), cesarean deliveries (0.58 ± 0.50) and abortions (0.51 ± 0.50). While most women did not have low symptoms or signs suggestive of thyroid hypofunction, they did have higher risk factor values (0.57) compared to the other clusters, such as age over 30 years (0.90 ± 0.31), autoimmune disease (0.09 ± 0.29) and history of abortion or preterm birth (0.44 ± 0.50). They had the second highest rate of diagnosis thyroid profile (0.25) and TX treatment (0.21). In addition, the cluster presented a higher number of patients with previous hypertension (0.08) and values of pregnancy complications, such as gestational diabetes (0.22), premature membrane rupture (0.21) and preterm delivery (0.16).

#### 3.2.4. Thyroid Pathology *k* = 2

The cluster analysis identified two women clusters. Table 6 shows the complete baseline data for the three prespecified features according to the cluster.

Cluster 0 (*n* = 56) had the lowest number of women and they mainly had higher risk factor values. These patients had the highest number of symptoms or signs suggestive a thyroid profile (0.75 ± 1.39). Furthermore, Cluster 0 women were more likely to be overweight (BMI of 25.43 ± 4.81), and half had presence of risk factors (0.52). Among the clusters, this one had the largest number of women diagnosed with a thyroid profile (0.39) and on TX treatment (0.38).

Cluster 1 (*n* = 250) was the largest, with more than three-quarters of all the subjects in our study. The women in this cluster had fewer symptoms or signs suggestive of thyroid hypofunction (0.31). Less women tended to be diagnosed with a thyroid profile (0.13) and to take TX treatment (0.12).

#### 3.2.5. Risk Factors *k* = 2

The cluster analysis identified two women clusters. Table 7 shows the complete baseline data for the 22 prespecified features according to the cluster.

Cluster 0 (*n* = 69) had the lowest number of women and all presented risk factors. Most of the subjects were likely to have almost two symptoms or signs suggestive of thyroid hypofunction (1.74 ± 1.31). The highest risk factors were fatigue (0.48) and constipation (0.41). Moreover, all patients had risk factors, such as goiter (0.03) and positive antibodies (0.06). These patients made up the largest number of women with thyroid profile (0.32 ± 0.47) and who followed a TX treatment (0.32 ± 0.47).

Cluster 1 (*n* = 237) was the largest, with more than two-thirds of all the subjects in our study. Importantly, all women in this cluster did not present symptoms or signs suggestive of thyroid hypofunction and the presence of risk factor was low (0.22 ± 0.41). They had a small number of diagnoses based on the thyroid profile (0.14 ± 0.35) and TX treatment (0.12 ± 0.33).

### 3.3. Clustering Prediction Using RF

To further investigate predictability in the current patient population, random forest classifier was carried out after cluster analysis. We performed the validation of different clusters using seven sets of features: (i) All features; (ii) risk factors; (iii) thyroid features; (iv) symptoms or signs suggestive of thyroid hypofunction; (v) all features without thyroid data; (vi) all features without perinatal, pregnancy and thyroid data; and (vii) all features without perinatal data and pregnancy. The RF accuracy is shown in Table 8.

There was a tendency to higher accuracy when using all the features, and observing the results the risk value had a high weight in the subgroups of features. (e) Risk factors computed 100.00% in all the sets beside the one composed for the thyroid features. In the case of (a) all features, the accuracy was perfect using the set of risk factors and computed the worst result using the thyroid features. For the features without perinatal and pregnancy features, (b) and (c), the computation differed depending on the number of clusters, *k* = 2 or *k* = 3. The error in the accuracy of (b) was due to a misclassification; Cluster 0 was computed as Cluster 1; (c) depends on the set of features, similar to the two clusters scenario, risk factors and all features; the misclassification was due to Cluster 0 being computed as Cluster 1. The rest of the sets misclassify in different clusters (i.e., Cluster 1 was predicted as Cluster 2 on four occasions; Cluster 2 was predicted as Cluster 0 on twelve occasions). (d) Thyroid pathology had a high accuracy when using all features, the thyroid features and all features without perinatal and pregnancy data.

## 4. Discussion

**Figure 2:**
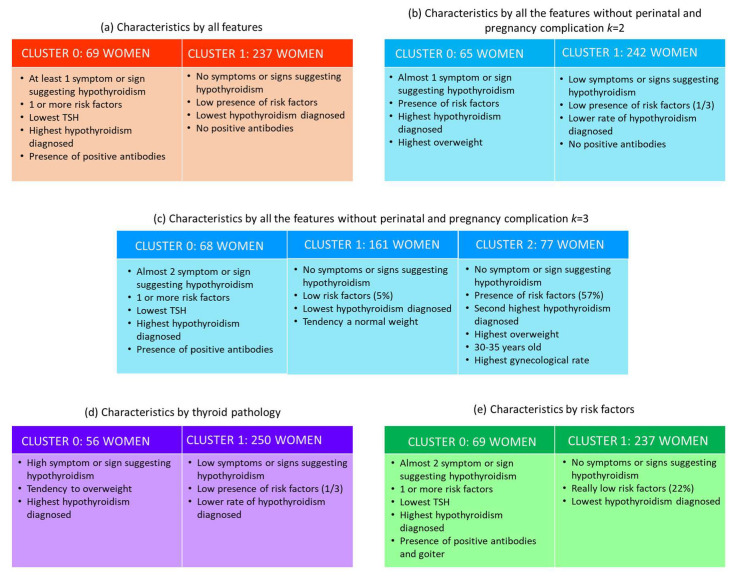
Cluster associations of women in first trimester of pregnancy with risk factors of thyroid pathology.

We applied a *k*-means clustering approach to a dataset from a recent large controlled trial of women in the first trimester of pregnancy to identify relevant phenotypes of thyroid pathology and risk factors. Women in each cluster varied considerably among several variables: Risk factors, age, weight and some pregnancy complications. We noted differential associations with risk factors and hypothyroidism (Figure 2). These findings underscore the significant heterogeneity that exists within the first trimester of pregnancy and the need for improved symptomatic phenotyping.

To our knowledge, this is the first application of cluster analysis to identify distinct risk factors and demographic features in a cohort of first-trimester pregnant women with a thyroid profile, a disorder believed to involve multiple disease subtypes [87,88,89,90,91]. Several previous studies analyzed the clinically relevant features and maternal outcomes of hypothyroid and euthyroid pregnancy, leading to new insights about the classification of women with similar patterns [92,93,94]. While the impact of these studies contributes to the literature, none of them focus on first trimester pregnancy or risk factor of thyroid pathology.

The findings presented here are important for several reasons, especially considering that there are only a handful of statistically identical characteristics in all subgroups; this emphasizes the need to improve descriptions of hypothyroidism in pregnancy subtypes. We identified two clusters of women using all features, features without perinatal results and pregnancy, thyroid pathology and risk factors. These groups were the result of hypothyroidism that differed in frequency and rates, especially in symptoms or signs suggesting hypothyroidism (prevalence: Almost 100% vs. almost 0%). Consequently, women in cluster 0 had a greater risk of fatigue, constipation, edema, positive antibodies and at least one symptom or sign suggesting hypothyroidism. Cluster 1 women seemed to be healthier, euthyroid and without risk factors and symptoms and signs that suggest hypothyroidism. According to the Organisation for Economic Co-operation and Development (OECD) [95], Mexico was the second most obese country of the 25 major economies, with 38.2% of Mexicans classified as obese, in 2015. Due to Mexico’s prevalence of overweight people and obesity, weight cannot be generalized to other populations and some comorbidities may not change between different clusters. Fatigue and constipation are common in both hypothyroidism and pregnancy. The number and combination of symptoms and signs may indicate a relationship with the diagnosis and treatment of hypothyroidism. Previous studies found a higher rate of 20–40% positive antibodies in hypothyroid pregnancy [94,96,97]. Despite the low rate of positive antibodies, new Mexican studies should be conduct for comparison. Treatment of overt hypothyroidism is recommended during pregnancy [3]. In this study, the positive autoimmune tests were not applied to all the women.

The use of the features without perinatal results and pregnancy with *k* = 3 gave additional information. Cluster 0 is represented by nearly two signs or symptoms related to hypothyroidism and has at least one risk factor, low pregnancy complications, a small TSH (6%) and a higher number of women diagnosed with hypothyroidism (32%). Cluster 1 was characterized by no symptoms or signs suggesting hypothyroidism and having low risk factors (5%); TSH was the same as the average population (12%) and the diagnosis of hypothyroidism was the lowest (9%). Cluster 2 had more women (16%) with TSH > 2.5 mU/L even with 25% previously diagnosed and in-treated. There are changes in thyroid function during pregnancy and usually previous doses are not enough to maintain optimal hormone levels. It is a changing hormonal system, so patients need to be monitored every few weeks and adjusted accordingly. In this study, a TSH blood test was performed during the first trimester for all pregnant patients that came for a prenatal care appointment (with and without treatment for hypothyroidism). Another reason is an increase in the TSH during pregnancy. The guidelines of the American Thyroid Association recommend monitoring TSH every four weeks until midgestation and at least once more around week 30 [3]. Subgroup analysis of maternal outcomes revealed a significant association of patients in this cluster with increased pregnancy complications, as the increase in patients with gestational diabetes (22%), premature membrane rupture (21%), preterm delivery (16%) and overweight women. Previous studies have associated hypothyroidism with preterm delivery [98,99,100]. Study [101] associated subclinical hypothyroidism with premature delivery at <20 weeks and [102] at <34 weeks. A high TSH in early pregnancy was associated with a risk of gestational diabetes [98,103] and premature membrane rupture [104,105]. A high TSH increased the risk of low birth weight [98,101]. In our study, this cluster birth weight was at least 100 g less than the others. Another study [106] concluded otherwise; for hypothyroidism they found higher weight at birth.

No significant association was found between baseline characteristics and preconception TSH clusters with most anthropometric, gynecological and perinatal outcomes. While untreated hypothyroidism can negatively affect pregnancy, there are no data suggesting that women with properly treated hypothyroidism are at increased risk for any obstetric complication [3].

Several limitations of this analysis require consideration. First and foremost, the present study was a retrospective analysis with T4 TOTAL, T4L and T3 not measured according to general recommendations, despite adequate diagnosis and treatment. Furthermore, the results of this study cannot be generalized to all pregnant populations as was done in northern Mexico. Likewise, the positive autoimmune test was not applied to the entire pregnant population since anti-TPO antibodies are not regularly requested due to their high cost to the patient. However, this study can be regarded as a baseline of the Mexican population and these results serve to majorly emphasize the need for a novel multidimensional classification of first trimester pregnant women with higher risk of thyroid disease, in order to improve patient care. We therefore recommend a larger prospective to assess the associations between maternal hypothyroidism and outcome features.

## 5. Conclusions

This study was carried out to explore the features in women during the first trimester of pregnancy using cluster analysis. This study recruited 306 patients during the first trimester of pregnancy reporting TSH values. Three distinct groups were identified using cluster analysis: (1) overweight women more than 30 years old who lacked signs or symptoms suggestive of thyroid hypofunction and a relatively low number of patients with some risk factors and increased pregnancy complications; (2) women less than 30 years old without any signs or symptoms suggestive of thyroid hypofunction, and who had both a low risk factor presence and had been diagnosed with a low level of hypothyroidism; (3) women less than 30 years old with a higher diagnosis of hypothyroidism that presents some risk factors and signs or symptoms suggestive of thyroid hypofunction; this group lacked autoimmune diseases and previous neck or head irradiation.

Cluster analysis was shown to be a practical approach for investigating the heterogeneity of the hypothyroidism risk factors in women in the first trimester of pregnancy in clinical studies. Risk factors and pregnancy complications might be valuable for prediction of hypothyroidism in pregnancy when compared with healthy patients. However, large-scale prospective trials with more information of pathological history, risk factors, thyroid pathology and pregnancy complications are necessary for further analysis.

## Figures and Tables

**Table 1:** Women in first trimester of pregnancy dataset.

Code	Variable	Conceptual Definition	Operational Definition	Data Type
ANTHROPOMETRIC				
Age	Age	Years born Patient age at the first trimester prenatal control consultation date	1 = 30>; 2 = 30–35; 3 = 35–40; 4 = 40<	Continuous
Weight	Weight	Weight measurement in kilograms (kg)	40.1–115.0	Continuous
Size	Height	Height in meters (m)	1.43–1.79	Continuous
BMI	BMI	It is defined as the weight of a person in kilograms divided by the square of his height in meters (k/m^2^)	15.4–43.8	Continuous
BMI-WHO	Body mass index - WHO classification	1 = Underweight if <18.5; 2 = normal weight if 18.5–24.9; 3 = overweight if 25–29.9; 4 = obese class I if 30-34.9; 5 = obese class II if 35–39.9; 6 = obese class III if > = 40 (k/m^2^)	1, 2, 3, 4, 5, 6	Categorical
GYNECOLOGICAL				
P	Pregnancies	Number of pregnancies	1–9	Discrete
D	Vaginal Deliveries	Number of vaginal deliveries	0, 1, 2, 3	Discrete
C	Caesarean Deliveries	Number of caesarean deliveries	0, 1, 2, 3, 4	Discrete
A	Abortions	Number of abortions	0, 1, 2, 3, 4, 5	Discrete
E	Ectopic	Number of ectopic pregnancies	0,1	Discrete
GW	GW	It is calculated using the LMP and the first ultrasound	4–13	Discrete
+ DAYS	+ DAYS	More days	0–9	Discrete
Total GW	Total GW	It is calculated using the LMP and the first ultrasound	4.0–13.86	Continuous
PATHOLOGICAL HISTORY				
CAH	Chronic Arterial Hypertension	Presence of chronic arterial hypertension	1 = yes; 0 = no	Binary
DM type II	DM type II	Presence of Diabetes Mellitus (DM) type II	1 = yes; 0 = no	Binary
Previous HTN	Previous HTN	Presence hypertensive disease in previous pregnancies	1 = yes; 0 = no	Binary
RISK FACTORS				
Age > 30	>30	Patients over 30 years	1 = yes; 0 = no	Binary
FHxTh	Family history of Thyroid Disease	Patients with a 1st family history of thyroid disease	1 = yes; 0 = no	Binary
ATD or Hypo T	Autoimmune Thyroid Disease or hypothyroidism	Patients with autoimmune thyroid disease or hypothyroidism	1 = yes; 0 = no	Binary
Goiter +	Goiter +	Presence of goiter	1 = yes; 0 = no	Binary
T+Anti TPO	T + Anti TPO	Presence of previous positive antibodies: anti-TPO, TRAb, and/or anti-thyroglobulin	0 = not performed, 1 = negative, 2 = positive	Categorical
Sx HypoT	SxHypoT	Patients with symptoms or signs suggestive of thyroid hypofunction.	1 = yes; 0 = no	Binary
Num Sx	NumSymptoms	Number of Symptoms or Signs Suggestive of Thyroid Hypofunction	0, 1, 2, 3, 4, 5	Discrete
FI	Fatigue	Symptom/sign suggestive of Th Hypofunction	1 = yes; 0 = no	Binary
CNST	Constipation	Symptom/sign suggestive of Th Hypofunction	1 = yes; 0 = no	Binary
Cold	Cold	Symptom/sign suggestive of Th Hypofunction	1 = yes; 0 = no	Binary
Myalgia	Myalgia	Symptom/sign suggestive of Th Hypofunction	1 = yes; 0 = no	Binary
+ weight	+ weight	Symptom/sign suggestive of Th Hypofunction	1 = yes; 0 = no	Binary
Edema	Edema	Symptom/sign suggestive of Th Hypofunction	1 = yes; 0 = no	Binary
Dry skin	Dry skin	Symptom/sign suggestive of Th Hypofunction	1 = yes; 0 = no	Binary
< Hair	Hair loss	Symptom/sign suggestive of Th Hypofunction	1 = yes; 0 = no	Binary
T1D	Diabetes T1	Presence of type I Diabetes	1 = yes; 0 = no	Binary
AD	Autoimmune disease	Autoimmune disease	1 = yes; 0 = no	Binary
Infertile	Infertile	Patients with a history of inability to achieve a clinical pregnancy after 12 months or more of unprotected sex according to WHO	1 = yes; 0 = no	Binary
IVF/ICSI	IVF/ICSI	In vitro fertilization/intracytoplasmic sperm injection	1 = yes; 0 = no	Binary
HxAB or PTB	History of Abortion or Preterm Birth	History of abortion or preterm birth	1 = yes; 0 = no	Binary
Prev. IRR	Prev. Irradiation neck or head	Prev. Irradiation neck or head	1 = yes; 0 = no	Binary
Ant. ThSurg	Ant. Thyroid Surgery	Anterior thyroid surgery	1 = yes; 0 = no	Binary
Current Tx with T4L	Current Tx with T4L	Patient undergoing replacement treatment with Levothyroxine	1 = yes; 0 = no	Binary
PresRF	Presence of some Risk Factor	Presence of any listed thyroid disease risk factor	1 = yes; 0 = no	Binary
THYROID PATHOLOGY				
TSH	TSH	TSH value reported in the first trimester Th profile	1 = 0.1 < TSH < 2.5 mU/L; 0 = normal	Real
T4 TOTAL	T4 TOTAL	T4 TOTAL value reported in the first trimester Th profile	1.89–14.63	Continuous
T4L	T4L	T4L value reported in the first trimester Th profile	0.5–8.88	Continuous
T3 TOTAL	T3 TOTAL	T3 TOTAL value reported in the first trimester Th profile	0.98–173.04	Continuous
DX Th	DX Thyroid Profile	Diagnosis based on the TSH and T4L values of the American Thyroid Association guidelines	1 = hypothyroidism; 0 = healthy	Binary
TX	TX	If treatment is indicated after obtaining thyroid profile results	1 = yes; 0 = no	Binary
PREGNANCY COMPLICATIONS				
HD with PG	Hypertensive Disease Associated with Pregnancy	Development of hypertensive disease from after the 20th week of pregnancy	1 = yes; 0 = no	Binary
GD	Gestational Diabetes	Impaired glucose levels detected during pregnancy according to the American Diabetes Association 2016	1 = yes; 0 = no	Binary
PROM	Premature Membrane Rupture	Rupture of amniotic membranes before the start of labor	1 = yes; 0 = no	Binary
PTD	Preterm Delivery	Childbirth that occurs before 37 weeks of gestation	1 = yes; 0 = no	Binary
PERINATAL RESULTS				
SDS	SDS at Unpacking	Weeks of Unemployment Gestation. It is calculated using the LMP and the first ultrasound.	29.1–41.2	Continuous
Birth	Birth Route	Birth Route	1 = delivery; 2 = caesarean	Binary
PW	Product weight	Measurement of the bodies in kilograms (kg)	1.190–4.385	Continuous
Ps	Product size	Height in centimeters (cm)	30–54	Continuous
Capurro	Capurro	Method to estimate the gestational age of a newborn	38–41	Discrete

**Table 2:** Number of women in the first trimester pregnancy clusters.

	0	1	2	3	4	5	6	7
**(a) All features**								
Cluster 2	69	237						
Cluster 3	71	167	68					
Cluster 4	61	162	40	43				
Cluster 5	47	45	15	161	38			
Cluster 6	36	81	59	89	39	2		
Cluster 7	51	78	47	3	14	33	80	
Cluster 8	48	90	11	3	46	36	68	4
**(b) All features without perinatal results and pregnancy complications**								
Cluster 2	65	241						
Cluster 3	77	161	68					
Cluster 4	62	107	42	95				
Cluster 5	45	51	43	160	7			
Cluster 6	63	122	62	3	23	33		
Cluster 7	49	99	19	3	98	2	36	
Cluster 8	41	156	17	8	6	33	41	4
**(c) Thyroid pathology**								
Cluster 2	56	250						
Cluster 3	56	28	222					
Cluster 4	28	56	26	196				
Cluster 5	27	196	9	48	26			
Cluster 6	27	196	9	32	26	16		
Cluster 7	27	188	16	31	26	9	9	
Cluster 8	27	188	9	30	26	16	9	1
**(d) Risk factors**								
Cluster 2	69	237						
Cluster 3	49	5	252					
Cluster 4	234	65	5	2				
Cluster 5	230	64	6	5	1			
Cluster 6	230	64	6	2	1	3		
Cluster 7	74	179	6	2	1	41	3	
Cluster 8	74	176	1	2	6	40	3	4

**Table 3:** Characteristics stratified by all features. Features are presented as mean ± SD.

Variable	Cluster 0 (*n* = 69)	Cluster 1 (*n* = 237)
ANTHROPOMETRIC		
Age	1.90 ± 0.73	1.87 ± 0.74
Weight	64.99 ± 11.57	64.40 ± 11.56
Height	1.61 ± 0.05	1.62 ± 0.06
BMI	24.99 ± 4.27	24.56 ±4.09
Body mass index - WHO classification	2.62 ± 0.79	2.48 ± 0.79
GYNECOLOGICAL		
Pregnancies	1.94 ± 1.04	2.19 ± 1.32
Vaginal Deliveries	0.10 ± 0.30	0.22 ± 0.41
Caesarean Deliveries	0.43 ± 0.50	0.42 ± 0.49
Abortions	0.22 ± 0.42	0.22 ± 0.41
Ectopic	0.01 ± 0.12	0.03 ± 0.16
GW	8.57 ± 2.65	8.21 ± 2.38
+ DAYS	2.43 ± 2.06	2.09 ± 2.10
Total GW	8.91 ± 2.68	8.51 ± 2.37
PATHOLOGICAL HISTORY		
DM type II	0.03 ± 0.17	0.01 ± 0.11
Previous HTN	0.01 ± 0.12	0.03 ± 0.16
RISK FACTORS		
>30	0.71 ± 0.46	0.70 ± 0.46
Family history of Thyroid Disease	0.03 ± 0.17	0.03 ± 0.16
Autoimmune Thyroid Disease or Hypothyroidism	0.03 ± 0.17	0.01 ± 0.11
Goiter +	0.03 ± 0.17	0.00 ± 0.00
T+Anti TPO	0.06 ± 0.24	0.00 ± 0.00
SxHipoT	0.99 ± 0.12	0.00 ± 0.00
NumSymptoms	1.74 ± 1.31	0.00 ± 0.00
Fatigue	0.48 ± 0.50	0.00 ± 0.00
Constipation	0.41 ± 0.49	0.00 ± 0.00
Cold	0.07 ± 0.26	0.00 ± 0.00
Myalgia	0.09 ± 0.28	0.00 ± 0.00
+ weight	0.12 ± 0.32	0.00 ± 0.00
Edema	0.16 ± 0.37	0.00 ± 0.00
Dry skin	0.04 ± 0.21	0.00 ± 0.00
Diabetes T1	0.00 ± 0.00	0.01 ± 0.09
Autoimmune disease	0.00 ± 0.00	0.03 ± 0.18
Infertile	0.09 ± 0.28	0.02 ± 0.13
IVF/ICSI	0.06 ± 0.24	0.01 ± 0.11
History of Abortion or Preterm Birth	0.17 ± 0.38	0.16 ± 0.37
Prev. Irradiation neck or head	0.00 ± 0.00	0.01 ± 0.09
Ant. Thyroid Surgery	0.01 ± 0.12	0.01 ± 0.09
Current Tx with T4L	0.04 ± 0.21	0.01 ± 0.11
Presence of some Risk Factor	1.00 ± 0.00	0.22 ± 0.41
THYROID PATHOLOGY		
TSH	0.06 ± 0.24	0.14 ± 0.34
DX Thyroid Profile	0.32 ± 0.47	0.14 ± 0.35
TX	0.32 ± 0.47	0.12 ± 0.33
PREGNANCY COMPLICATIONS		
Hypertensive Disease Associated with Pregnancy	0.06 ± 0.24	0.02 ± 0.13
Gestational Diabetes	0.10 ± 0.30	0.15 ± 0.36
Premature Membrane Rupture	0.03 ± 0.17	0.08 ± 0.27
Preterm Delivery	0.04 ± 0.21	0.06 ± 0.24
PERINATAL RESULTS		
SDS at Unpacking	38.54 ± 1.31	38.40 ± 1.50
Birth Route	1.70 ± 0.46	1.73 ± 0.44
Product Weight	3150.42 ± 487.36	3131.15 ± 448.78
Product size	48.96 ± 1.77	48.77 ± 2.39

**Table 4:** Characteristics stratified by all the features without perinatal and pregnancy complication results. Features are presented as mean ± SD.

Variable	Cluster 0 (*n* = 65)	Cluster 1 (*n* = 241)
ANTHROPOMETRIC		
Age	1.80 ± 0.73	1.90 ± 0.73
Weight	68.40 ± 13.50	63.49 ± 10.76
Height	1.62 ± 0.05	1.62 ± 0.06
BMI	25.96 ± 4.81	24.30 ± 3.83
Body mass index - WHO classification	2.74 ± 0.96	2.45 ± 0.73
GYNECOLOGICAL		
Pregnancies	2.26 ± 1.50	2.12 ± 1.20
Vaginal Deliveries	0.12 ± 0.33	0.21 ± 0.41
Caesarean Deliveries	0.52 ± 0.50	0.39 ± 0.49
Abortions	0.29 ± 0.46	0.20 ± 0.40
Ectopic	0.00 ± 0.00	0.03 ± 0.17
GW	9.03 ± 2.60	8.09 ± 2.37
+ DAYS	2.32 ± 2.16	2.13 ± 2.07
Total GW	9.36 ± 2.60	8.39 ± 2.37
PATHOLOGICAL HISTORY		
DM type II	0.03 ± 0.17	0.01 ± 0.11
Previous HTN	0.05 ± 0.21	0.02 ± 0.13
RISK FACTORS		
>30	0.65 ± 0.48	0.71 ± 0.45
Family history of Thyroid Disease	0.03 ± 0.17	0.02 ± 0.16
Autoimmune Thyroid Disease or Hypothyroidism	0.05 ± 0.21	0.01 ± 0.09
Goiter +	0.03 ± 0.17	0.00 ± 0.00
T+Anti TPO	0.03 ± 0.17	0.01 ± 0.09
SxHipoT	0.42 ± 0.50	0.17 ± 0.38
NumSymptoms	0.85 ± 1.31	0.27 ± 0.78
Fatigue	0.22 ± 0.41	0.08 ± 0.27
Constipation	0.12 ± 0.33	0.08 ± 0.28
Cold	0.05 ± 0.21	0.01 ± 0.09
Myalgia	0.06 ± 0.24	0.01 ± 0.09
+ weight	0.09 ± 0.29	0.01 ± 0.09
Edema	0.09 ± 0.29	0.02 ± 0.14
Dry skin	0.05 ± 0.21	0.00 ± 0.00
Diabetes T1	0.02 ± 0.12	0.00 ± 0.06
Autoimmune disease	0.03 ± 0.17	0.02 ± 0.16
Infertile	0.09 ± 0.29	0.02 ± 0.13
IVF/ICSI	0.05 ± 0.21	0.02 ± 0.13
History of Abortion or Preterm Birth	0.18 ± 0.39	0.16 ± 0.37
Prev. Irradiation neck or head	0.00 ± 0.00	0.01 ± 0.09
Ant. Thyroid Surgery	0.03 ± 0.17	0.00 ± 0.06
Current Tx with T4L	0.06 ± 0.24	0.01 ± 0.09
Presence of some Risk Factor	0.58 ± 0.50	0.34 ± 0.47
THYROID PATHOLOGY		
TSH	0.12 ± 0.33	0.12 ± 0.32
DX Thyroid Profile	0.38 ± 0.49	0.12 ± 0.33
TX	0.37 ± 0.49	0.11 ± 0.32
PREGNANCY COMPLICATIONS		
Hypertensive Disease Associated with Pregnancy	0.05 ± 0.21	0.02 ± 0.14
Gestational Diabetes	0.12 ± 0.33	0.14 ± 0.35
Premature Membrane Rupture	0.05 ± 0.21	0.07 ± 0.26
Preterm Delivery	0.08 ± 0.27	0.05 ± 0.22
PERINATAL RESULTS		
SDS at Unpacking	38.21 ± 1.29	38.49 ± 1.49
Birth Route	1.72 ± 0.45	1.73 ± 0.45
Product Weight	3144.89 ± 420.96	3132.96 ± 467.06
Product size	48.57 ± 2.16	48.88 ± 2.29

**Table 5:** Characteristics stratified by all features. Features are presented as mean ± SD.

Variable	Cluster 2 (*n* = 68)	Cluster 1 (*n* = 161)	Cluster 0 (*n* = 77)
ANTHROPOMETRIC			
Age	1.87 ± 0.69	1.69 ± 0.62	2.29 ± 0.82
Weight	65.08 ± 11.63	62.34 ± 9.26	68.63 ± 14.41
Height	1.61 ± 0.05	1.62 ± 0.06	1.62 ± 0.05
BMI	25.01 ± 4.30	23.84 ± 3.39	26.04 ± 4.95
Body mass index - WHO classification	2.63 ± 0.79	2.35 ± 0.65	2.73 ± 1.00
GYNECOLOGICAL			
Pregnancies	1.90 ± 0.98	1.73 ± 0.77	3.19 ± 1.66
Vaginal Deliveries	0.09 ±0.29	0.18 ± 0.39	0.30 ± 0.46
Caesarean Deliveries	0.43 ± 0.50	0.34 ± 0.48	0.58 ± 0.50
Abortions	0.22 ± 0.42	0.07 ± 0.26	0.51 ± 0.50
Ectopic	0.01 ± 0.12	0.01 ± 0.11	0.05 ± 0.22
GW	8.57 ± 2.67	7.98 ± 2.41	8.68 ± 2.25
+ DAYS	2.46 ± 2.07	2.14 ±2.20	1.97 ± 1.86
Total GW	2.92 ± 2.70	8.29 ± 2.41	8.96 ± 2.23
PATHOLOGICAL HISTORY			
DM type II	0.03 ± 0.17	0.00 ± 0.00	0.04 ± 0.19
Previous HTN	0.01 ± 0.12	0.00 ± 0.00	0.08 ± 0.27
RISK FACTORS			
>30	0.71 ± 0.46	0.60 ± 0.49	0.90 ± 0.31
Family history of Thyroid Disease	0.03 ± 0.17	0.03 ± 0.17	0.01 ± 0.11
Autoimmune Thyroid Disease or Hypothyroidism	0.03 ± 0.17	0.00 ± 0.00	0.04 ± 0.19
Goiter +	0.03 ± 0.17	0.00 ± 0.00	0.00 ± 0.00
T+Anti TPO	0.06 ± 0.24	0.00 ± 0.00	0.00 ± 0.00
SxHipoT	0.99 ± 0.12	0.00 ± 0.00	0.01 ± 0.11
NumSymptoms	1.75 ± 1.32	0.00 ± 0.00	0.01 ± 0.11
Fatigue	0.47 ± 0.50	0.00 ± 0.00	0.01 ± 0.11
Constipation	0.41 ± 0.50	0.00 ± 0.00	0.00 ± 0.00
Cold	0.07 ± 0.26	0.00 ± 0.00	0.00 ± 0.00
Myalgia	0.09 ± 0.29	0.00 ± 0.00	0.00 ± 0.00
+ weight	0.12 ± 0.32	0.00 ± 0.00	0.00 ± 0.00
Edema	0.16 ± 0.37	0.00 ± 0.00	0.00 ± 0.00
Dry skin	0.04 ± 0.21	0.00 ± 0.00	0.00 ± 0.00
Diabetes T1	0.00 ± 0.00	0.00 ± 0.00	0.03 ± 0.16
Autoimmune disease	0.00 ± 0.00	0.01 ± 0.08	0.09 ± 0.29
Infertile	0.09 ± 0.29	0.01 ± 0.08	0.04 ± 0.19
IVF/ICSI	0.06 ± 0.24	0.00 ± 0.00	0.04 ± 0.19
History of Abortion or Preterm Birth	0.18 ± 0.38	0.03 ± 0.17	0.44 ± 0.50
Prev. Irradiation neck or head	0.00 ± 0.00	0.00 ± 0.00	0.03 ± 0.16
Ant. Thyroid Surgery	0.01 ± 0.12	0.00 ± 0.00	0.03 ± 0.16
Current Tx with T4L	0.04 ± 0.21	0.00 ± 0.00	0.04 ± 0.19
Presence of some Risk Factor	1.00 ± 0.00	0.05 ± 0.22	0.57 ± 0.50
THYROID PATHOLOGY			
TSH	0.06 ± 0.24	0.12 ± 0.33	0.16 ± 0.37
DX Thyroid Profile	0.32 ± 0.47	0.09 ± 0.28	0.25 ± 0.43
TX	0.32 ± 0.47	0.08 ± 0.27	0.21 ± 0.41
PREGNANCY COMPLICATIONS			
Hypertensive Disease Associated with Pregnancy	0.06 ± 0.24	0.02 ± 0.14	0.01 ± 0.11
Gestational Diabetes	0.10 ± 0.31	0.11 ± 0.32	0.22 ± 0.42
Premature Membrane Rupture	0.01 ± 0.12	0.02 ± 0.16	0.21 ± 0.41
Preterm Delivery	0.03 ± 0.17	0.02 ± 0.14	0.16 ± 0.37
PERINATAL RESULTS			
SDS at Unpacking	38.62 ± 1.13	38.74 ± 0.98	37.63 ± 2.12
Birth Route	1.69 ± 0.47	1.71 ± 0.45	1.78 ± 0.42
Product Weight	3172.19 ± 455.93	3166.80 ± 406.39	3037.63 ± 542.46
Product size	49.05 ± 1.63	49.06 ± 1.78	48.07 ± 3.29

**Table 6:** Characteristics stratified by thyroid pathology. Features are presented as mean ± SD.

Variable	Cluster 0 (*n* = 56)	Cluster 1 (*n* = 250)
ANTHROPOMETRIC		
Age	1.84 ± 0.73	1.89 ± 0.74
Weight	66.95 ± 13.35	63.99 ± 11.06
Height	1.62 ± 0.05	1.62 ± 0.06
BMI	25.43 ± 4.81	24.48 ± 3.95
Body mass index - WHO classification	2.64 ± 0.92	2.48 ± 0.76
GYNECOLOGICAL		
Pregnancies	2.38 ± 1.58	2.08 ± 1.18
Vaginal Deliveries	0.14 ± 0.35	0.20 ± 0.40
Caesarean Deliveries	0.54 ± 0.50	0.40 ± 0.49
Abortions	0.32 ± 0.47	0.19 ± 0.39
Ectopic	0.00 ± 0.00	0.03 ± 0.17
GW	9.02 ± 2.54	8.12 ± 2.40
+ DAYS	2.39 ± 2.21	2.12 ± 2.06
Total GW	9.36 ± 2.53	8.43 ± 2.40
PATHOLOGICAL HISTORY		
DM type II	0.04 ± 0.19	0.01 ± 0.11
Previous HTN	0.04 ± 0.19	0.02 ± 0.14
RISK FACTORS		
>30	0.68 ± 0.47	0.70 ± 0.46
Family history of Thyroid Disease	0.04 ± 0.19	0.02 ± 0.15
Autoimmune Thyroid Disease or Hypothyroidism	0.05 ± 0.23	0.01 ± 0.09
Goiter +	0.02 ± 0.13	0.00 ± 0.06
T+Anti TPO	0.02 ± 0.13	0.01 ± 0.11
SxHipoT	0.32 ± 0.47	0.20 ± 0.40
NumSymptoms	0.75 ± 1.39	0.31 ± 0.81
Fatigue	0.16 ± 0.37	0.10 ± 0.30
Constipation	0.13 ± 0.33	0.08 ± 0.28
Cold	0.05 ± 0.23	0.01 ± 0.09
Myalgia	0.04 ± 0.19	0.02 ± 0.13
+ weight	0.05 ± 0.23	0.02 ± 0.14
Edema	0.05 ± 0.23	0.03 ± 0.18
Dry skin	0.05 ± 0.23	0.00 ± 0.00
Diabetes T1	0.02 ± 0.13	0.00 ± 0.06
Autoimmune disease	0.04 ± 0.19	0.02 ± 0.15
Infertile	0.07 ± 0.26	0.02 ± 0.15
IVF/ICSI	0.04 ± 0.19	0.02 ± 0.14
History of Abortion or Preterm Birth	0.20 ± 0.40	0.16 ± 0.37
Prev. Irradiation neck or head	0.00 ± 0.00	0.01 ± 0.09
Ant. Thyroid Surgery	0.02 ± 0.13	0.01 ± 0.09
Current Tx with T4L	0.07 ± 0.26	0.01 ± 0.09
Presence of some Risk Factor	0.52 ± 0.50	0.36 ± 0.48
THYROID PATHOLOGY		
TSH	0.14 ± 0.35	0.11 ± 0.32
DX Thyroid Profile	0.39 ± 0.49	0.13 ± 0.34
TX	0.38 ± 0.49	0.12 ± 0.33
PREGNANCY COMPLICATIONS		
Hypertensive Disease Associated with Pregnancy	0.05 ± 0.23	0.02 ± 0.14
Gestational Diabetes	0.09 ± 0.29	0.15 ± 0.36
Premature Membrane Rupture	0.05 ± 0.23	0.07 ± 0.26
Preterm Delivery	0.07 ± 0.26	0.05 ± 0.22
PERINATAL RESULTS		
SDS at Unpacking	38.21 ± 1.33	38.48 ± 1.48
Birth Route	1.70 ± 0.46	1.73 ± 0.44
Product Weight	3137.59 ± 408.52	3135.03 ± 467.94
Product size	48.58 ± 2.32	48.86 ± 2.25

**Table 7:** Characteristics stratified by risk factors. Features are presented as mean ± SD.

Variable	Cluster 0 (*n* = 69)	Cluster 1 (*n* = 237)
ANTHROPOMETRIC		
Age	1.90 ± 0.73	1.87 ± 0.74
Weight	64.99 ± 11.57	64.40 ± 11.56
Height	1.61 ± 0.05	1.62 ± 0.06
BMI	24.99 ± 4.27	24.56 ±4.09
Body mass index - WHO classification	2.62 ± 0.79	2.48 ± 0.79
GYNECOLOGICAL		
Pregnancies	1.94 ± 1.04	2.19 ± 1.32
Vaginal Deliveries	0.10 ± 0.30	0.22 ± 0.41
Caesarean Deliveries	0.43 ± 0.50	0.42 ± 0.49
Abortions	0.22 ± 0.42	0.22 ± 0.41
Ectopic	0.01 ± 0.12	0.03 ± 0.16
GW	8.57 ± 2.65	8.21 ± 2.38
+ DAYS	2.43 ± 2.06	2.09 ± 2.10
Total GW	8.91 ± 2.68	8.51 ± 2.37
PATHOLOGICAL HISTORY		
DM type II	0.03 ± 0.17	0.01 ± 0.11
Previous HTN	0.01 ± 0.12	0.03 ± 0.16
RISK FACTORS		
>30	0.71 ± 0.46	0.70 ± 0.46
Family history of Thyroid Disease	0.03 ± 0.17	0.03 ± 0.16
Autoimmune Thyroid Disease or Hypothyroidism	0.03 ± 0.17	0.01 ± 0.11
Goiter +	0.03 ± 0.17	0.00 ± 0.00
T+Anti TPO	0.06 ± 0.24	0.00 ± 0.00
SxHipoT	0.99 ± 0.12	0.00 ± 0.00
NumSymptoms	1.74 ± 1.31	0.00 ± 0.00
Fatigue	0.48 ± 0.50	0.00 ± 0.00
Constipation	0.41 ± 0.49	0.00 ± 0.00
Cold	0.07 ± 0.26	0.00 ± 0.00
Myalgia	0.09 ± 0.28	0.00 ± 0.00
+ weight	0.12 ± 0.32	0.00 ± 0.00
Edema	0.16 ± 0.37	0.00 ± 0.00
Dry skin	0.04 ± 0.21	0.00 ± 0.00
Diabetes T1	0.00 ± 0.00	0.01 ± 0.09
Autoimmune disease	0.00 ± 0.00	0.03 ± 0.18
Infertile	0.09 ± 0.28	0.02 ± 0.13
IVF/ICSI	0.06 ± 0.24	0.01 ± 0.11
History of Abortion or Preterm Birth	0.17 ± 0.38	0.16 ± 0.37
Prev. Irradiation neck or head	0.00 ± 0.00	0.01 ± 0.09
Ant. Thyroid Surgery	0.01 ± 0.12	0.01 ± 0.09
Current Tx with T4L	0.04 ± 0.21	0.01 ± 0.11
Presence of some Risk Factor	1.00 ± 0.00	0.22 ± 0.41
THYROID PATHOLOGY		
TSH	0.06 ± 0.24	0.14 ± 0.34
DX Thyroid Profile	0.32 ± 0.47	0.14 ± 0.35
TX	0.32 ± 0.47	0.12 ± 0.33
PREGNANCY COMPLICATIONS		
Hypertensive Disease Associated with Pregnancy	0.06 ± 0.24	0.02 ± 0.13
Gestational Diabetes	0.10 ± 0.30	0.15 ± 0.36
Premature Membrane Rupture	0.03 ± 0.17	0.08 ± 0.27
Preterm Delivery	0.04 ± 0.21	0.06 ± 0.24
PERINATAL RESULTS		
SDS at Unpacking	38.54 ± 1.31	38.40 ± 1.50
Birth Route	1.70 ± 0.46	1.73 ± 0.44
Product Weight	3150.42 ± 487.36	3131.15 ± 448.78
Product size	48.96 ± 1.77	48.77 ± 2.39

**Table 8:** RF accuracy using seven sets of features.

Features	(a) All Features	(b) No Perinatal and Pregnancy *k* = 2	(c) No Perinatal and Pregnancy *k* = 3	(d) Thyroid Pathology	(e) Risk Factors
All features	100.00%	97.78%	92.71%	98.91%	100.00%
Risk factors	100.00%	80.61%	87.75%	72.04%	100.00%
Thyroid features	81.00%	97.53%	55.68%	97.67%	76.53%
Symptoms or signs suggestive of thyroid hypofunction	98.94%	77.22%	80.58%	76.92%	100.00%
All features without thyroid data	98.60%	77.43%	93.13%	75.90%	100.00%
All features without perinatal, pregnancy and thyroid data	98.23%	76.84%	88.54%	74.07%	100.00%
All features without perinatal and pregnancy data	98.91%	79.85%	94.38%	97.06%	100.00%

## Supplementary Materials

The following are available online at https://www.mdpi.com/2077-0383/9/7/2247/s1, Table S1: Classification according to thyroid pathology, Table S2: Features presented with the mean ± SD.
