# Supplementary material for: Cluster Analysis: A New Approach for Identification of Underlying Risk Factors and Demographic Features of First Trimester Pregnancy Women
Source: J Clin Med. 2020 Jul 15;9(7):2247. doi: 10.3390/jcm9072247 (PMC7408845; doi:10.3390/jcm9072247)
Supplement: Supplementary file 1 [file jcm-09-02247-s001.pdf]

Supplementary Material

The prevalence of Thyroid Pathology is presented in Table S1 with the classification related to euthyroid, subclinical hypothyroidism and frank hypothyroidism.

Table S1 Classification according to thyroid pathology.

<table>
<tr><th colspan="2"></th><th>Total
n (%)</th></tr>
<tr><td rowspan="3">Classification related to Thyroid Profile</td><td>Euthyroid</td><td>251 (82%)</td></tr>
<tr><td>Subclinical hypothyroidism</td><td>36 (11.8%)</td></tr>
<tr><td>Overt hypothyroidism</td><td>19 (6.2%)</td></tr>
<tr><td colspan="2">Total</td><td>306 (100%)</td></tr>
</table>

The results of the variables used in this study is presented in Table S2.

Table S2. Features presented with the mean ± SD

Variable	All Data
ANTHROPOMETRIC	
Age	1.88 ± 0.73
Weight	64.53 ± 11.55
Height	1.62 ± 0.06
BMI	24.65 ± 4.13
Body mass index - WHO classification	2.51 ± 0.79
GYNECOLOGICAL	
Pregnancies	2.14 ± 1.26
Vaginal Deliveries	0.19 ± 0.39
Caesarean Deliveries	0.42 ± 0.49
Abortions	0.22 ± 0.41
Ectopic	0.02 ± 0.15
GW	8.29 ± 2.44
+ DAYS	2.17 ± 2.09
Total GW	8.60 ± 2.45
PATHOLOGICAL HISTORY	
DM type II	0.02 ± 0.13
Previous HTN	0.02 ± 0.15
RISK FACTORS	
>30	0.70 ± 0.46
Family history of Thyroid Disease	0.03 ± 0.16
Autoimmune Thyroid Disease or Hypothyroidism	0.02 ± 0.13
Goiter +	0.01 ± 0.08
T+Anti TPO	0.01 ± 0.11
SxHipoT	0.22 ± 0.42
NumSymptoms	0.39 ± 0.96
Fatigue	0.11 ± 0.31
Constipation	0.09 ± 0.29
Cold	0.02 ± 0.13
Myalgia	0.02 ± 0.14
+ weight	0.03 ± 0.16
Edema	0.04 ± 0.19
Dry skin	0.01 ± 0.10
Diabetes T1	0.01 ± 0.08
Autoimmune disease	0.03 ± 0.16
Infertile	0.03 ± 0.18
IVF/ICSI	0.02 ± 0.15

History of Abortion or Preterm Birth	0.17 ± 0.37
Prev. Irradiation neck or head	0.01 ± 0.08
Ant. Thyroid Surgery	0.01 ± 0.10
Current Tx with T4L	0.02 ± 0.14
Presence of some Risk Factor	0.39 ± 0.49
THYROID PATHOLOGY	
TSH	0.12 ± 0.32
DX Thyroid Profile	0.18 ± 0.38
TX	0.17 ± 0.37
PREGNANCY COMPLICATIONS	
Hypertensive Disease Associated with Pregnancy	0.03 ± 0.16
Gestational Diabetes	0.14 ± 0.34
Premature Membrane Rupture	0.07 ± 0.25
Preterm Delivery	0.06 ± 0.23
PERINATAL RESULTS	
SDS at Unpacking	38.43 ± 1.46
Birth Route	1.73 ± 0.45
Product Weight	3135.50 ± 457.01
Product size	48.81 ± 2.26
